# Bone marrow mesenchymal stem cell-derived exosomal lncRNA KLF3-AS1 stabilizes Sirt1 protein to improve cerebral ischemia/reperfusion injury via miR-206/USP22 axis

**DOI:** 10.1186/s10020-022-00595-1

**Published:** 2023-01-10

**Authors:** Xiaowei Xie, Yu Cao, Liangping Dai, Dingzhou Zhou

**Affiliations:** 1 Department of Comprehensive Surgery, Hunan Provincial People’s Hospital (The First-Affiliated Hospital of Hunan Normal University), Changsha, 410005 Hunan People’s Republic of China; 2 Clinical Nursing Teaching and Research Section, The Second Xiangya Hospital, Central South University, Changsha, 410011 Hunan People’s Republic of China

**Keywords:** BMSC-Exos, KLF3-AS1, Cerebral I/R, Neuroinflammation, miR-206, USP22, Sirt1

## Abstract

**Background:**

Cerebral ischemia/reperfusion (I/R) is a pathological process that occurs in ischemic stroke. Bone marrow mesenchymal stem cell-derived exosomes (BMSC-Exos) have been verified to relieve cerebral I/R-induced inflammatory injury. Hence, we intended to clarify the function of BMSC-Exos-delivered lncRNA KLF3-AS1 (BMSC-Exos KLF3-AS1) in neuroprotection and investigated its potential mechanism.

**Methods:**

To mimic cerebral I/R injury in vivo and in vitro, middle cerebral artery occlusion (MCAO) mice model and oxygen–glucose deprivation (OGD) BV-2 cell model were established. BMSC-Exos KLF3-AS1 were administered in MCAO mice or OGD-exposed cells. The modified neurological severity score (mNSS), shuttle box test, and cresyl violet staining were performed to measure the neuroprotective functions, while cell injury was evaluated with MTT, TUNEL and reactive oxygen species (ROS) assays. Targeted genes and proteins were detected using western blot, qRT-PCR, and immunohistochemistry. The molecular interactions were assessed using RNA immunoprecipitation, co-immunoprecipitation and luciferase assays.

**Results:**

BMSC-Exos KLF3-AS1 reduced cerebral infarction and improved neurological function in MCAO mice. Similarly, it also promoted cell viability, suppressed apoptosis, inflammatory injury and ROS production in cells exposed to OGD. BMSC-Exos KLF3-AS1 upregulated the decreased Sirt1 induced by cerebral I/R. Mechanistically, KLF3-AS1 inhibited the ubiquitination of Sirt1 protein through inducing USP22. Additionally, KLF3-AS1 sponged miR-206 to upregulate USP22 expression. Overexpression of miR-206 or silencing of Sirt1 abolished KLF3-AS1-mediated protective effects.

**Conclusion:**

BMSC-Exos KLF3-AS1 promoted the Sirt1 deubiquitinating to ameliorate cerebral I/R-induced inflammatory injury via KLF3-AS1/miR-206/USP22 network.

**Supplementary Information:**

The online version contains supplementary material available at 10.1186/s10020-022-00595-1.

## Introduction

Ischemic stroke remains the leading causes of morbidity and mortality around the world (Anthony et al. 2022). Despite significant advances in recanalization strategies, patients are still at risk for cerebral ischemia/reperfusion (I/R) injury (Tuo et al. 2022), in which neuroinflammation is significantly involved (Jurcau and Simion 2021). Cerebral I/R injury induces the release of pro-inflammatory cytokines, such as interleukin 6 (IL-6), monocyte chemotactic protein-1 (MCP-1) and tumor necrosis factor alpha (TNFα) (Zhang et al. 2021a, b), and inflammatory mediator reactive oxygen species (ROS) (Chen and Li 2020), which aggravates brain injury and contributes to the pathological process of cerebral I/R injury. Nevertheless, the deep understanding of inflammatory cascade reaction during cerebral I/R injury is still urgently needed to be further explored for developing effective interventions.

Exosomes are small vesicles with a diameter of 30–150 nm and release from most cells in body (Rastogi et al. 2021). Exosomes carry numerous molecules, such as proteins, mRNAs and other RNA species, which are thought to be of paramount importance for intercellular communication (Li et al. 2021). For treating ischemic stroke, stem cells-secreted exosomes could be a prospective therapeutic choice due to the unique properties, including low immunogenicity and stable biological characteristic (Xiong et al. 2022). Plenty of researches have also implied that bone marrow mesenchymal stem cell-derived exosomes (BMSC-Exos) might supply a therapeutic strategy for relieving neuroinflammation after cerebral I/R injury (Liu et al. 2021).

Long non-coding RNAs (lncRNAs), longer than 200 nucleotides, are a kind of non-coding RNAs that do not encode proteins (Wolska et al. 2021). The significant role of lncRNAs has become to be emphasized in cerebral I/R injury (Ghafouri-Fard et al. 2020). LncRNA KLF3-AS1 in exosomes derived from mesenchymal stem cells (MSC) has been reported to improve myocardial infarction (Mao et al. 2019). However, whether MSC-derived exosomes carry KLF3-AS1 to participate in cerebral I/R damage remains indistinct. Current investigations have revealed that the competing endogenous RNA (ceRNA) networks mediated by lncRNAs play a vital role in cerebral I/R damage (Zhang et al. 2021a, b). The potential binding sequences of KLF3-AS1 and microRNA(miR)-206 has been forecasted with bioinformatics. Inhibition of miR-206 ameliorates I/R arrhythmia in a mouse model (Jin et al. 2020). Moreover, MSC-derived exosomal KLF3-AS1 inhibit chondrocytes apoptosis by sponging miR-206 in osteoarthritis (Liu et al. 2018). Silent mating type information regulation 2 homolog 1 (Sirt1) is a nicotine adenine dinucleotide (NAD)-dependent enzyme (Zhang et al. 2018). Sirt1 has been proved to play a neuroprotective role in cerebral ischemia by the anti-inflammatory properties (Jiao and Gong 2020). For example, the upregulated Sirt1 induced by cycloastragenol represses apoptosis and neuroinflammation following brain ischemia (Li et al. 2020). Given that MSC-released exosomal KLF3-AS1 regulates Sirt1 in osteoarthritis (Mao et al. 2019), whether MSC-derived exosomal KLF3-AS1 regulates Sirt1 in cerebral I/R injury need to be further explored. Furthermore, previous studies have revealed that Sirt1 ubiquitination can be modulated by ubiquitin specific peptidase 22 (USP22), mouse double minute 2 homolog (MDM2) and c-Jun N-terminal kinase (JNK) proteins (Gao et al. 2011; Peng et al. 2015; Xiong et al. 2017). Especially, upregulation of USP22 can remove the conjugated ubiquitin chain of Sirt1, therefore reducing the degradation of Sirt1 protein mediated by ubiquitin in hepatocellular carcinoma (Xiong et al. 2017); A previous study has revealed that USP22-mediated Sirt1 signaling is involved in the tumorigenesis (Xu et al. 2018); Moreover, the binding sites of miR-206 with USP22 have been forecasted by bioinformatics. Whether KLF3-AS1 regulates Sirt1 via miR-206/USP22 axis is unclear in cerebral I/R injury.

We hypothesized that MSC exosomal KLF3-AS1 promoted Sirt1 stability to ameliorate cerebral I/R induced-inflammatory injury through KLF3-AS1/miR-206/USP22 network. In this study, the neuroprotective role of MSC exosomal KLF3-AS1 were initially examined in a cerebral I/R mice model. The effects and potential mechanism of MSC exosomal KLF3-AS1 were then investigate in an in vitro model of cerebral I/R.

## Materials and methods

### Isolation and characterization of BMSC

The tibia and femora were collected from C57BL/6 mice (male, 8 weeks old) for the isolation of primary BMSC. In brief, mice were anesthetized by ether before sacrificed. Next, after sterilized with 75% ethanol, tibia and femora were dissected. Dulbecco’s Modified Eagle Medium (DMEM, Gibco, Grand Island, NY, USA) was applied to flush the marrow cavities. To harvest BMSC, the insolated cells in the suspension were centrifuged at 1000 rpm (5 min). The obtained cells were maintained in DMEM with 1% penicillin/streptomycin and 10% fetal bovine serum (FBS, Gibco) in a humidified incubator at 37 °C with 5% CO_2_. The cells were sub-cultured every 3 days. At the third generation, cells were collected and evaluated with flow cytometry (Becton Dickinson, Franklin Lakes, NJ, USA) for identifying the typical positive and negative BMSC markers, including HLA-DR (Invitrogen, Carlsbad, CA, USA), CD105 (BD Biosciences, San Jose, CA, USA), CD34 (BD Biosciences), CD90 (BD Biosciences) and CD45 (BD Biosciences). BMSC at passages 3 to 8 were employed for the following studies.

### Isolation and characterization of BMSC-Exos

Cell culture supernatants of BMSC at a density of 1 × 10^6^ were harvested to isolate BMSC-Exos by serial differential centrifugation combined with filter. Briefly, the supernatants were first centrifuged for 30 min at 2000*g*, followed by centrifuged for 30 min at 10,000*g*. After filtered through a 0.22 μm strainer (Millipore, Bedford, MA, USA), the supernatants were centrifuged at 4 °C for 2 h at 100,000*g*. These isolated exosomes were rinsed with PBS and resuspended. Exosomes in the supernatant of BMSC transfected with KLF3-AS1 overexpression vector or control vector were also isolated, according to the above methods. Three kinds of BMSC-Exos were got: Exos, Exos-vector and Exos-KLF3-AS1. Total proteins were quantified using a BCA protein assay kit (Thermo). Finally, 20 μL exosomes was added onto a copper grid for 60 s at room temperature. After wiped the redundant solution, 2% phosphotungstic acid solution (30 μL, pH = 6.8) was added for another 60 s. An incandescent light was used to roast the grid for 10 min. Then the morphology of the exosomes was observed under transmission electron microscopy (TEM). The diameter of exosomes was examined by Nanoparticle Tracking Analysis (NTA; Particle Metrix, Inning am Ammersee, Germany). Exosome markers, including CD63 (Abcam, Cambridge, MA, USA), CD81 (Abcam), CD9 (Abcam) and TSG101 (Abcam), were tested by western blot. 5 μg of protein from exosomes was applied for western blot analysis.

### Cell culture and oxygen–glucose deprivation (OGD) treatment

BV-2 murine microglial cells acquired from BeNa Culture Collection (Beijing, China) were maintained at 37 °C in DMEM added with 1% penicillin/streptomycin and 10% FBS in humidified atmosphere with 5% CO_2_. BV-2 cells were subjected to OGD for simulating cerebral I/R damage in vitro. Briefly, BV-2 cells were maintained in glucose-free DMEM for 2 h at 37 °C, with 94% N_2_, 1% O_2_ and 5% CO_2_. Afterwards, cells were cultured in normal DMEM under normoxic condition for 24 h at 37 °C. Cells in control group were maintained under normal condition. For in vitro experiments, 10 μg/ml BMSC-Exos was added and incubated in BV-2 cells.

### Cell transfection

The pcDNA3.1-KLF3-AS1, miR-206 mimic, short hairpin RNAs (shRNAs) targeting Sirt1 (sh-Sirt1) or USP22 (sh-USP22), and corresponding negative controls were obtained from GenePharma. Cells (3 × 10^5^) were planted into a 6-well plate for 24 h, followed by transfected with the indicated plasmids (50 nM) by Lipofectamine 2000 reagent (Invitrogen). Cycloheximide (10 μg/ml, Sigma) was added into cells and incubated for 0, 2, 4, 6 or 8 h to block protein synthesis. To block proteasome activity, cells were administrated with MG132 (10 μM, Sigma) for 12 h.

### MTT assay

Cell viability was detected by MTT assay. BV-2 cells were planted in a 96-well plate followed by corresponding treatments. Then each well was added with MTT (20 μL, Sigma) before incubation at 37 °C for 4 h. To resolve MTT formazan crystals, each well was added with 150 μL DMSO (Sigma) after the culture medium was removed. A microplate reader (Bio-Rad, Hercules, CA, USA) was applied for measuring the absorbance at 490 nm.

### TUNEL assay

Apoptotic cells were tested by TUNEL assay. In brief, cells were fixed in 4% paraformaldehyde after washed by PBS. Next, they were incubated with TUNEL reaction mixture including labeling solution and enzyme solution, in the dark overnight at 4 ℃. After stained by DAPI (Abcam) for 5 min, cells were examined under fluorescence microscopy (Olympus, Tokyo, Japan) for obtaining the images. In 6 regions of each cell slide, the number of DAPI-positive cells and TUNEL/DAPI double-positive cells were counted for calculating the proportion of TUNEL-positive cells.

### ROS assay

Cells were planted in 6-well plates prior to corresponding treatments. They were added with 2′,7′-dichlorofluoresceindiacetate (CM2-DCFHDA, 5 μM, Invitrogen) at 37 °C for 30 min. After washed by PBS, cells were fixed for 20 min in 4% formaldehyde. Next, they were stained by DAPI (Abcam) and analyzed by fluorescence microscopy (Olympus).

### qRT-PCR analysis

Trizol reagent (Invitrogen) was used to extract total RNA, and Nanodrop2000 (Thermo Fisher, Waltham, MA, USA) was applied to measure RNA purity and concentration. By employing PrimeScript™ RT kit (Thermo), RNA samples were transcribed reversely into cDNA. A 7500 FAST Real-Time PCR System (Applied Biosystems, Foster City, CA, USA) was applied for the PCR amplification with SYBR Premix Ex Taq II (Thermo). GAPDH and U6 were selected as internal controls. The relative expression levels of RNA were calculated by 2^−ΔΔCt^ method. The primers are presented in Table 1.

**Table 1:** The primer sequences used in qRT-PCR

Gene	Forward primer (5’-3’)	Reversed primer (5’-3’)
KLF3-AS1	AATGAGTGCGTGGAGGAAAT	CCTGGGCAACAGAGTGAGAC
MCP-1	GCAGGTCCCTGTCATGCTTC	ACAGCTTCTTTGGGACACCT
TNFα	GACGTGGAACTGGCAGAAGAG	TTGGTGGTTTGTGAGTGTGAG
IL-6	GGTCCAGTTGCCTTCTCCC	GCAACAAGGAACACCACGG
Sirt1	GCTGACGACTTCGACGACG	TCGGTCAACAGGAGGTTGTCT
USP22	CCATTGATCTGATGTACGGAGG	TCCTTGGCGATTATTTCCATGTC
miR-185-5p	GCGCGATTGGAGAGAAAGGCAGT	ATCCAGTGCAGGGTCCGAGG
miR-138-5p	CCCAGGGTCTGGTGCGGAGA	CAGGGGCTGAGCGGTGAGGG
miR-206	GGAATGTAAGGAAGTGTGTG	GAACATGTCTGCGTATCTC
miR-23c	CCAGAAGGACGTAGAAG	CTTCACTGTGATGGGCTC
miR-223	GGCAGCACCCCATAAACTGTT	CAGTGCGTGTCGTGTCGTGGAG
U6	GCTTCGGCAGCACATATACTAAAAT	CGCTTCACGAATTTGCGTGTCAT
GAPDH	AGGTCGGTGTGAACGGATTTG	TGTAGACCATGTAGTTGAGGTCA

### Western blot

Brain tissues, cells or exosomes were harvested at the indicated times. Protein lysates were prepared with RIPA buffer (Beyotime) containing protease inhibitor cocktail (Thermo). BCA protein assay kit (Beyotime) was used for protein concentration detection. Proteins were removed onto PVDF membrane (Millipore) after isolated in SDS-PAGE (10%). 5% non-fat milk was applied to block the membrane for 1 h. Then the membrane was incubated in primary antibodies overnight at 4 °C. Primary antibodies anti-IL-6, anti-TNFα and anti-MCP-1 were acquired from Santa Cruz, while anti-Sirt1, anti-USP22, anti-MDM2, anti-p-JNK and anti-β-actin were obtained from Abcam. Following incubating in secondary antibodies conjugated with HRP (Thermo), the blots were then visualized by ECL reagent (Beyotime).

### RNA immunoprecipitation (RIP)

The Magna RNA-binding protein immunoprecipitation kit (Millipore) was used to perform RIP assay. In brief, RIP buffer containing magnetic beads conjugated by anti-Ago2 (Abcam) or normal IgG was added into cell lysates and incubated overnight at 4 °C. The immunoprecipitated RNA was isolated from the samples by incubation with proteinase K. Afterwards, qRT-PCR was performed to analysis the purified RNAs.

### Dual luciferase reporter assay

KLF3-AS1 was predicted to bind with miR-206 in starBase. The binding sites of miR-206 with USP22 were forecasted in TargetScan software. On the bases of the forecasted binding sites, mutant (mut) and wild (wt) sequences (mut-KLF3-AS1, wt-KLF3-AS1, mut-Sirt1 and wt-Sirt1) were synthesized, then the sequences were cloned into luciferase reporter vectors (pmir-GLO, Promega, Madison, WI, USA). BV-2 cells were planted in a 24-well plate, prior to co-transfection with luciferase reporter vector (100 ng) and miR-206 mimics (30 nM, GenePharma) with lipofectamine 2000 reagent (Invitrogen). Following 24 h transfection, dual luciferase reporter assay system (Promega) was used to detect the luciferase activity.

### Co-immunoprecipitation

Co-immunoprecipitation assay was conducted using an immunoprecipitation kit (Abcam) as per the manufacturer's recommendations. In brief, BV-2 cells were transfected for 24 h with sh-USP22 or pcDNA3.1-KLF3-AS1. Cell lysate was prepared using RIPA buffer (Thermo). Each sample was incubated with Sirt1 antibody (Abcam) or USP22 antibody (Abcam) at 4 °C overnight. Afterwards, protein A/G Sepharose beads were added before incubated for 4 h at 4 °C. Protein-bead complex was eluted with NuPAGE LDS Sample Buffer (Life Technologies). The immunoprecipitated fractions were detected using western blot with anti-Sirt1 (Abcam), anti-ubiquitin (Abcam), anti-USP22 (Abcam) or anti-Tubulin (Abcam).

### Middle cerebral artery occlusion (MCAO) in mice

C57BL/6J mice (8 weeks old, male) were acquired from Hunan Provincial People’s Hospital (The first-affiliated hospital of Hunan normal university). All animals were housed with free access to water and food in a controlled environment. After mice anesthetized with pentobarbital sodium, a midline cervical incision was made to expose the left common carotid artery of mice. To occlude the origin of the middle cerebral artery, the left internal carotid artery was inserted into a nylon filament-coated thread. After 2 h, the thread was withdrawn for reperfusion. Mice in sham group only received vascular separation without occlusion. Then 4 groups of MCAO mice with similar mNSS were included, namely MCAO, Exos (50 μg), Exos-vector (50 μg), Exos-KLF3-AS1 (50 μg) groups (n = 5 per group). At day 2 and day 14 after MCAO, mice were injected intravenously with BMSC-Exos (200 μL in PBS/mice) via tail vein. An equal volume of PBS was administrated in sham and MCAO mice. Mice were sacrificed at day 28 after the surgery. The study procedures were agreed by the Animal Care and Use Committee in Hunan Provincial People’s Hospital (The first-affiliated hospital of Hunan normal university).

### Behavioral tests

Behavioral tests were performed in mice to assess the neurological function. The modified neurological severity score (mNSS) was carried out at the indicated time points following MCAO. The mNSS involves several tests to evaluate sensory, motor, balance and reflex abilities. Neurological dysfunction was more severe with a higher score, with a maximum value of 18. On the last three days, shuttle box test was performed. In Brief, the apparatus consisted of two connected compartments. Prior to the final test, the mice were adapted to the apparatus for two days. For the training trial, mice might move to the opposite compartment when the light, sound or electricity stimulation was delivered, otherwise another unconditioned stimulus would deliver. For the test trial, the frequency and response time of active avoidance of the mice were observed to assess the learning and memory capabilities. Finally, after excessively anesthetized, mice were subjected to cardiac perfusion with cold saline prior to fixed with 4% paraformaldehyde. Brain tissues were collected for the next analysis.

### Infarction volume assessment

Brain tissues were coronally cut down into slices (2 mm thick). To calculate the infarct volume, 0.1% cresyl violet solution was added and stained the sections. For eliminating the influence of brain edema, infarct size was determined according to the formula: Infarction volume % = (contralateral hemisphere volume – non-lesioned volume of ipsilateral hemisphere)/ contralateral hemisphere volume × 100%.

### Immunohistochemistry staining

Frozen coronal Sects. (25 μm) of the brain tissues were prepared. Following incubation for 1 h in donkey serum (5%), sections were administrated in anti-Sirt1 (Abcam) at 4 °C overnight. Next, biotinylated secondary antibody (Invitrogen) was added and incubated for 30 min at 37 °C. Antibody-antigen complexes were visualized after the addition of fresh diaminobenzidine (Beyotime). A microscope (Olympus) was applied to acquire the images.

### Statistical analysis

Statistical analysis was performed using SPSS17.0. Figures are presented as means ± SD. The data between two groups was analyzed applying Student’s t-test, while one-way ANOVA were applied for comparisons among three or more groups. P < 0.05 was considered as statistical significance.

## Results

### Exosomal KLF3-AS1 protected against cerebral I/R damage in a mice model

**Fig. 1:**
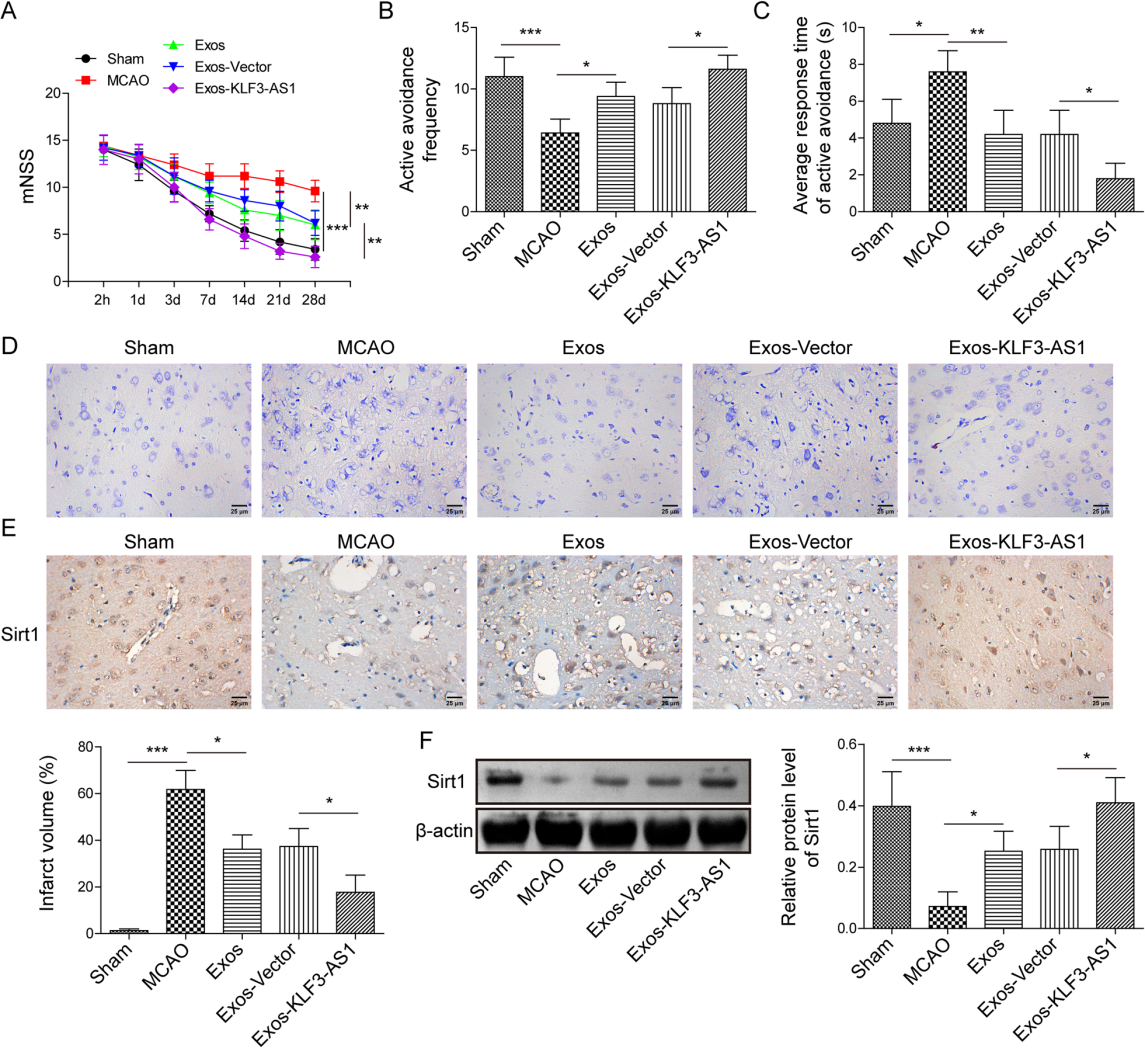
The role of Exos-KLF3-AS1 in MCAO mice. Mice were subjected to MCAO surgery, followed by injected intravenously with sdyExos, Exos-vector or Exos-KLF3-AS1. **A** Neurological severity scores of mice. **B**, **C** The active avoidance behavior of mice by shuttle box test. **D** Infarct volume was calculated. Sirt1 expression detected by immunohistochemistry (**E**) and western blot (**F**). n = 5 per group. Data are described as mean ± SD. *P < 0.05, **P < 0.01, ***P < 0.001

First, BMSC and exosomes were isolated. Our results indicated that BMSC was negative for HLA-DR, CD45 and CD34 but positive for CD105 and CD90 (Additional file 1: Fig. S1A). Exosomes derived from BMSC presented a round-shaped morphology, demonstrated by TEM (Additional file 1: Fig. S1B). The diameter of exosomes was about 30—120 nm, indicated by NTA (Additional file 1: Fig. S1C). In addition, BMSC-Exos were positive for TSG101, CD9 and CD81, which were the specific exosome surface markers, while negative for CD9, showed by western blot (Additional file 1: Fig. S1D). Taken together, the data demonstrated the effective isolation of BMSC-Exos. KLF3-AS1 was highly expressed in BMSC-Exos, compared to that in BMSC (Additional file 1: Fig. S1E), suggesting that BMSC-Exos were particularly rich in KLF3-AS1. Then BMSC were transfected with KLF3-AS1 overexpression vector, and KLF3-AS1 expression in exosomes isolated from BMSC was measured by qRT-PCR, which indicated the successful overexpression of KLF3-AS1 both in BMSC and BMSC-Exos (Additional file 1: Fig. S1F and G). To further verify the biological functions of exosomal KLF3-AS1, MCAO mice were treated with Exos, Exos-vector or Exos-KLF3-AS1. Except Sham group, neurological impairment was observed in all mice 2 h after MCAO, besides, mNSS presented a persistent decrease thereafter. Compared to MCAO, Exo or Exos-Vector groups, the score in the Exos-KLF3-AS1 group was decreased from Day 7 to Day 28 (Fig. 1A), suggesting that exosomal KLF3-AS1 improved MCAO-induced neurological dysfunction. Next, an active avoidance test was carried out for evaluating learning and memory deficits. The active avoidance frequency of Exos-KLF3-AS1 group was increased in comparison with MCAO, Exo or Exos-Vector groups (Fig. 1B). Compared with MCAO group, the average response time of active avoidance in Exos-KLF3-AS1 group was downregulated, but no significant difference was observed between MCAO and Exo or Exos-Vector groups (Fig. 1C). Significant cerebral infarction was observed in MCAO mice, furthermore, compared to that in MCAO, Exo or Exos-Vector groups, the infarct volume in Exo-KLF3-AS1 group was reduced (Fig. 1D), indicating that exosomal KLF3-AS1 could optimally alleviate cerebral I/R injury in vivo. Sirt1 protein level was reduced in MCAO mice; importantly, compared to that in MCAO group, Sirt1 protein levels in Exo, Exos-Vector or Exo-KLF3-AS1 groups were all increased, especially in Exo-KLF3-AS1 group (Fig. 1E, F).

### The protective role of exosomal KLF3-AS1 in a cell model of cerebral I/R injury

**Fig. 2:**
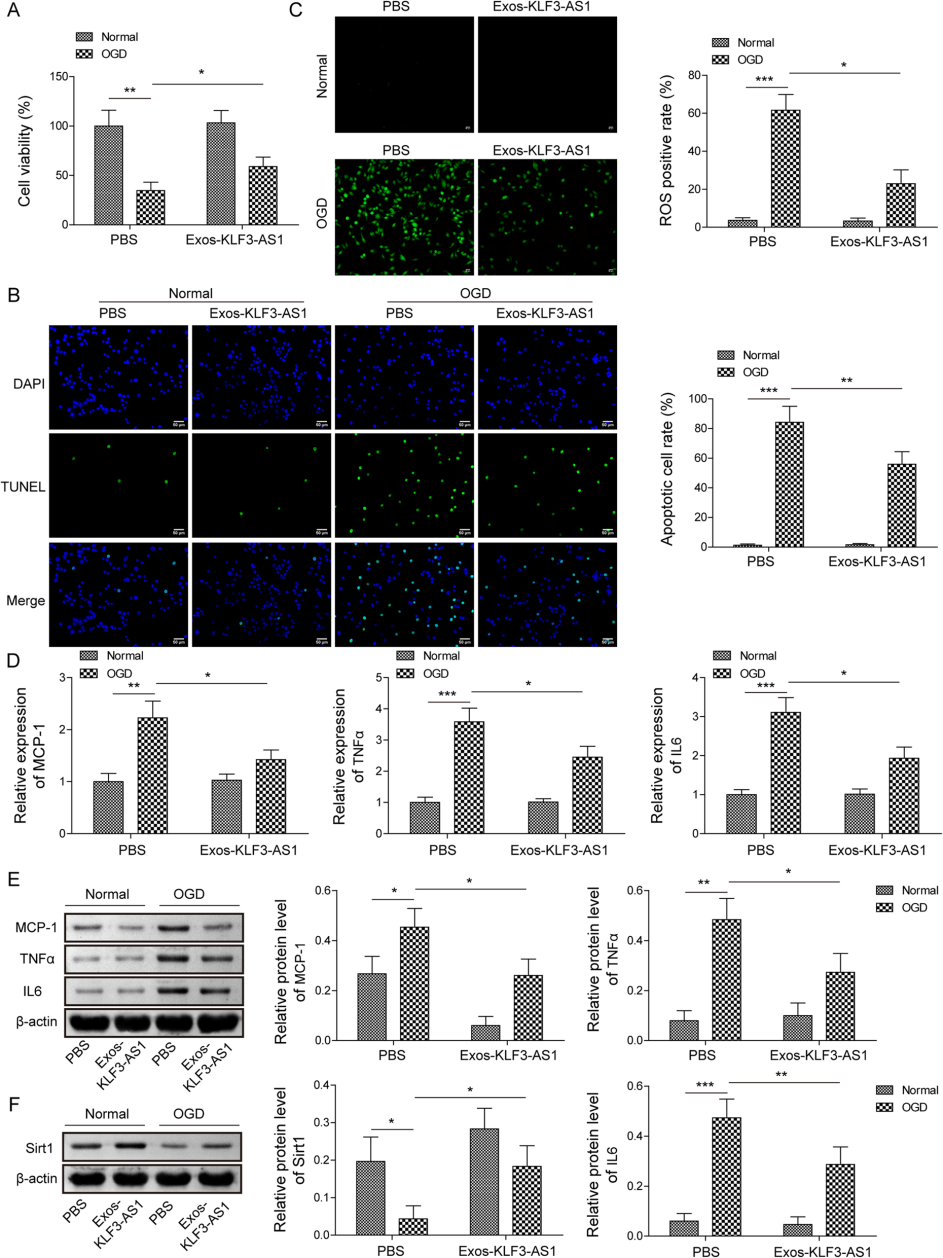
The role of Exos-KLF3-AS1 in OGD exposed cells in vitro. Cells were subjected to OGD followed by treated with PBS or Exos-KLF3-AS1. **A** Cell viability determined by MTT. **B** Cell apoptosis assessed with TUNEL assay. **C** ROS production determined by ROS assay. **D**, **E** The mRNA and protein levels of MCP-1, TNFα and IL-6 detected using qRT-PCR and western bolt. **F** Sirt1 protein expression detected by western bolt. Values were expressed as mean ± SD of three separate determinations in the in vitro assays. *P < 0.05, **P < 0.01, ***P < 0.001

For validating the biological roles of exosomal KLF3-AS1, BV-2 cells subjected to OGD were treated with Exos-KLF3-AS1. Cell viability in OGD-subjected cells was declined in comparison with control group, whereas Exos-KLF3-AS1 rescued the declined cell viability induced by OGD (Fig. 2A). Cell apoptosis was increased in OGD-treated cells, while Exos-KLF3-AS1 alleviated OGD-induced cell apoptosis (Fig. 2B). The production of ROS was increased in OGD-treated cells, which was repressed by the supplement of Exos-KLF3-AS1 (Fig. 2C). OGD exposure upregulated the mRNA and protein levels of inflammatory factors (TNFα, IL-6 and MCP-1), whereas Exos-KLF3-AS1 mitigated the inflammatory injury induced by OGD (Fig. 2D, E). These results suggested that exosomal KLF3-AS1 attenuated cerebral I/R injury in vitro. Furthermore, compared to control group, Sirt1 protein was decreased in OGD-exposed cells, which was upregulated by the addition of Exos-KLF3-AS1 (Fig. 2F).

### KLF3-AS1 upregulated Sirt1 protein via enhancing its stability

**Fig. 3:**
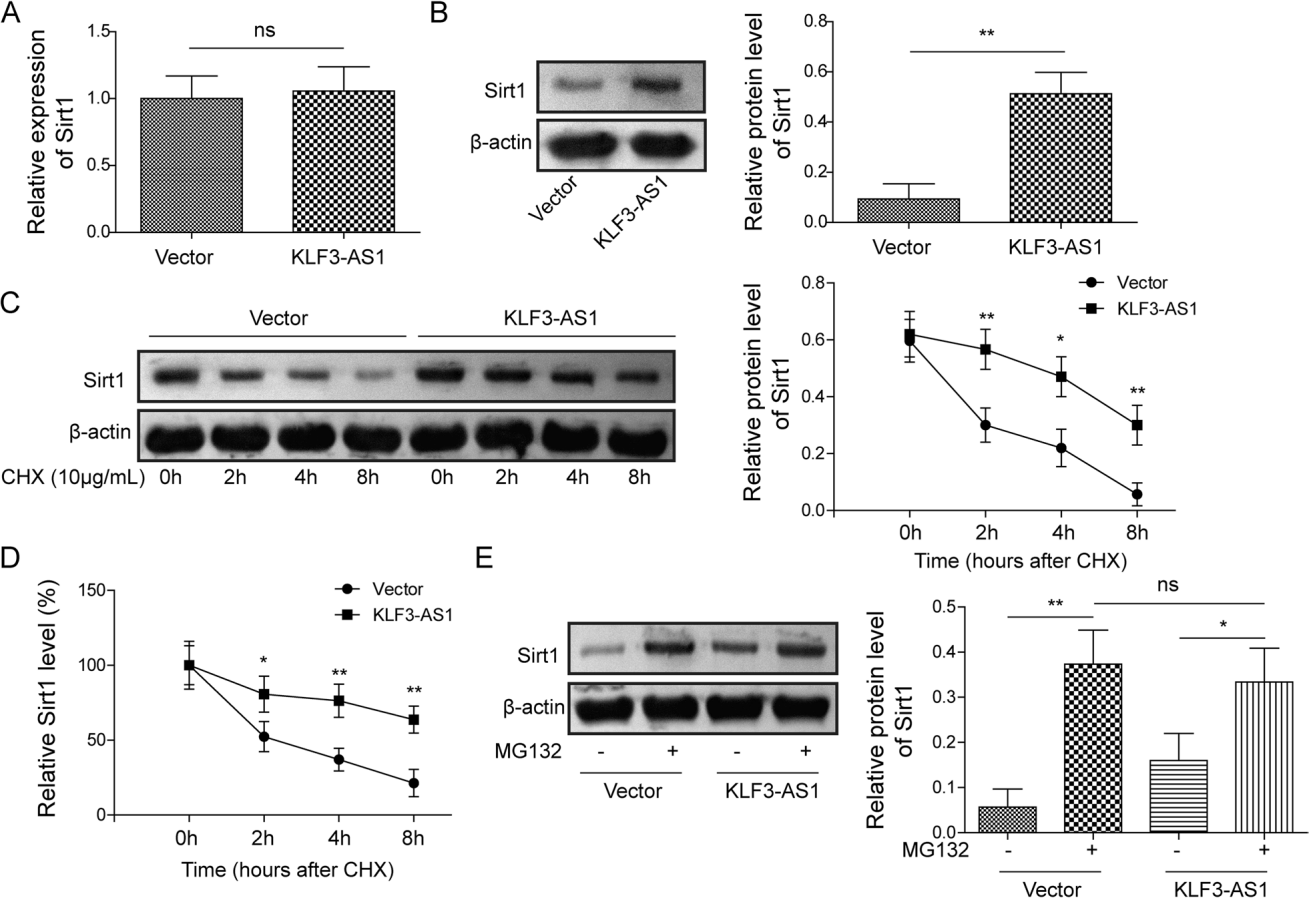
The effect of KLF3-AS1 on Sirt1 protein expression and its stability. **A**, **B** Cells were treated by vector or KLF3-AS1 overexpression plasmid. Sirt1 mRNA and protein levels were determined. **C**, **D** Cells transfected with KLF3-AS1 overexpression plasmid or vector were treated with cycloheximide, and Sirt1 protein level were detected. **E** After transfected with KLF3-AS1 overexpression plasmid or vector, Cells were treated with MG132. And Sirt1 protein level was detected. Values were expressed as mean ± SD of three separate determinations in the in vitro assays. *P < 0.05, **P < 0.01, ***P < 0.001

We then illustrated how Sirt1 expression was affected by KLF3-AS1. The result revealed that Sirt1 mRNA expression was not substantially altered in cells with KLF3-AS1 overexpression (Fig. 3A). However, Sirt1 protein level was upregulated by KLF3-AS1 overexpression (Fig. 3B). KLF3-AS1’ effect on Sirt1 degradation was next analyzed. Cells transfected with or without KLF3-AS1 overexpression plasmid were treated by cycloheximide for the indicated time duration, then the remaining Sirt1 was measured. The decreased level of Sirt1 was restrained by KLF3-AS1 transfection, compared to that in Vector group (Fig. 3C, D). The data revealed that KLF3-AS1 inhibited the degradation of Sirt1 protein. Moreover, the degradation of Sirt1 protein could be rescued in both Vector and KLF3-AS1 groups with the addition of proteasome inhibitor MG132 (Fig. 3E), suggesting that KLF3-AS1 upregulated Sirt1 protein level by inhibiting Sirt1 degradation.

### KLF3-AS1 induced USP22 to promote the deubiquitination of Sirt1 protein

**Fig. 4:**
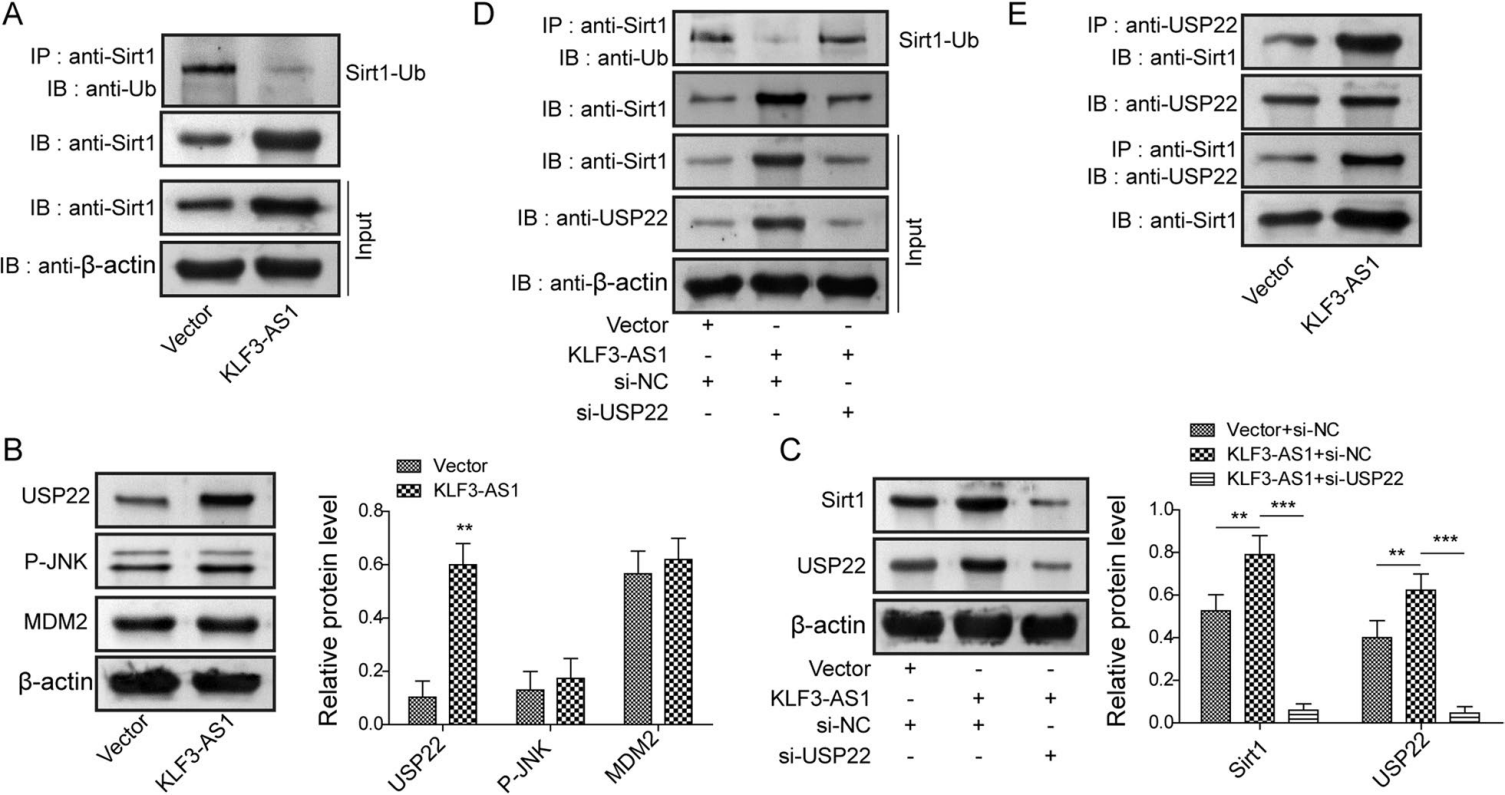
USP22 silencing antagonized the effect of KLF3-AS1 on Sirt1 deubiquitination. **A**, **B** BV-2 cells were transfected with KLF3-AS1 overexpression plasmid or vector, Sirt1 ubiquitination was evaluated by Co-immunoprecipitation (**A**), then protein expressions of USP22, MDM2 and P-JNK were tested (**B**). Cells were transfected with pcDNA-KLF3-AS1 or sh-USP22, then Sirt1 and USP22 proteins were detected by western bolt (**C**), and Sirt1 deubiquitination was assessed by Co-immunoprecipitation (**D**). **E** Cells were transfected with KLF3-AS1 overexpression plasmid or vector, the interaction between USP22 and Sirt1 was then validated by Co-immunoprecipitation. Values were expressed as mean ± SD of three separate determinations in the in vitro assays. **P < 0.01, ***P < 0.001

Afterwards, the role of KLF3-AS1 on Sirt1 ubiquitination was explored. The data from Co-immunoprecipitation demonstrated that KLF3-AS1 overexpression promoted the deubiquitination of Sirt1 (Fig. 4A). Sirt1 ubiquitination can be modulated by USP22, MDM2 and JNK proteins (Gao et al. 2011; Peng et al. 2015; Xiong et al. 2017). Our data showed that USP22 protein level was upregulated after overexpression of KLF3-AS1, while MDM2 and P-JNK were not obviously altered (Fig. 4B), indicating that USP22 might be involved in mediating the ubiquitination of Sirt1. Furthermore, overexpression of KLF3-AS1 upregulated Sirt1 and USP22, but silencing of USP22 reversed these effects (Fig. 4C). Knockdown of USP22 reversed the deubiquitination of Sirt1 mediated by KLF3-AS1 (Fig. 4D). By using Co-immunoprecipitation, the interaction between USP22 and Sirt1 was validated in cells transfected with KLF3-AS1 overexpression plasmid or not (Fig. 4E). Taken together, KLF3-AS1 induced USP22 expression to promote the deubiquitination of Sirt1 protein.

### KLF3-AS1 functioned as a ceRNA by sponging miR-206 to upregulate USP22

**Fig. 5:**
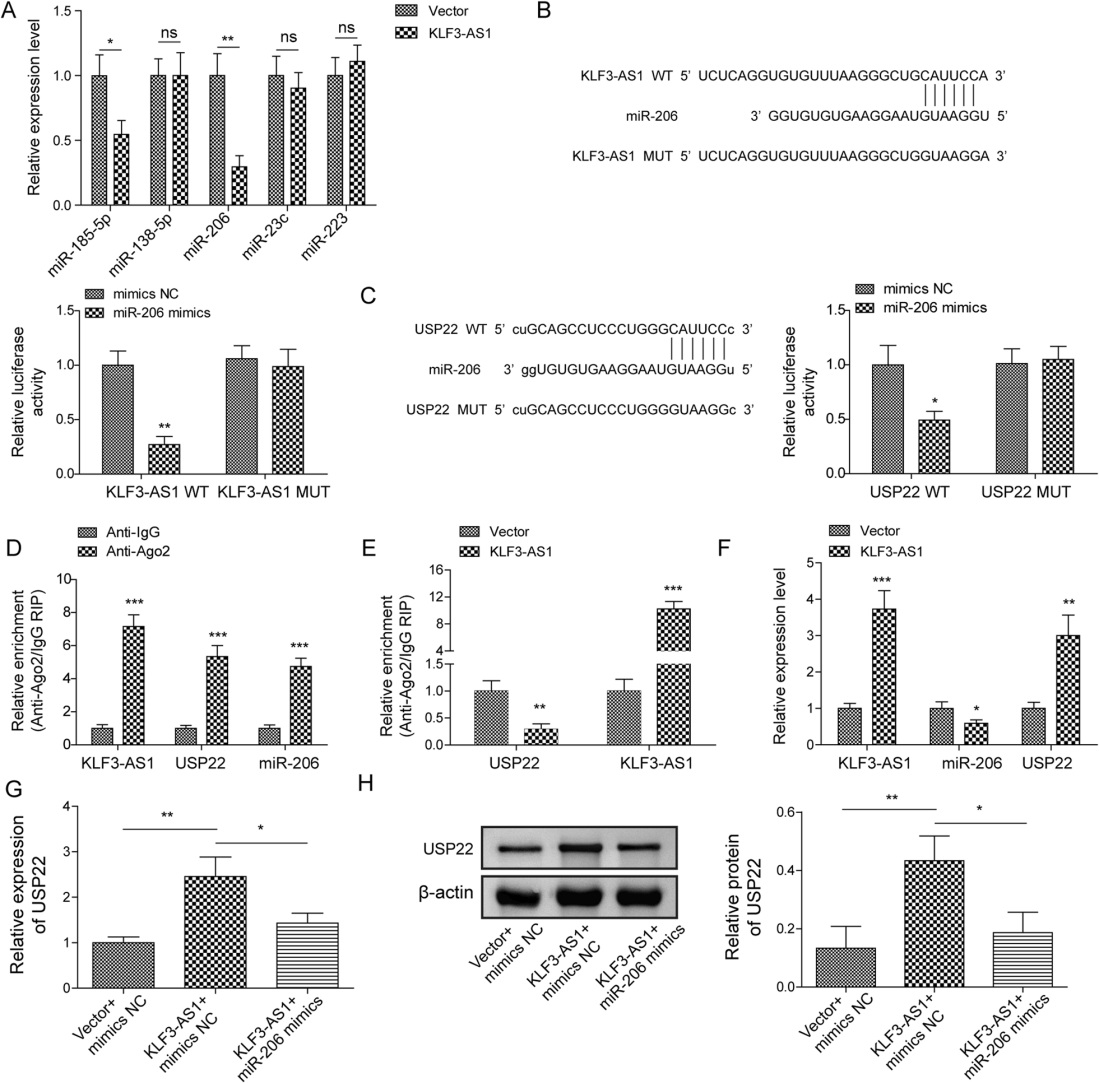
The interaction of KLF3-AS1, miR-206and USP22. **A** Bioinformatics predicted the binding sites of several miRNAs with KLF3-AS1. Cells were transfected with pcDNA-KLF3-AS1 or control vector, then miRNAs levels were detected. **B**, **C** Luciferase activity examined using luciferase reporter assay. **D** Anti-Ago2 RIP assay in BV-2 cells was performed, then KLF3-AS1, miR-206 and USP22 mRNA associated with Ago2 were measured. **E** Cells were transfected with pcDNA-KLF3-AS1, RIP assay was then carried out. And the enrichment of KLF3-AS1 and USP22 was detected. **F** KLF3-AS1, miR-206 and USP22 mRNA levels were measured in cells transfected with pcDNA-KLF3-AS1. **G**, **H** Cells were transfected with KLF3-AS1 overexpression plasmid, vector or miR-206 mimic, then qRT-PCR (**G**) or western blot (**H**) was performed for measuring USP22. Values were expressed as mean ± SD of three separate determinations in the in vitro assays. *P < 0.05, **P < 0.01, ***P < 0.001

The molecular mechanism of KLF3-AS1 in regulating USP22 was then further investigated. Bioinformatics predicted several miRNAs, including miR-185-5p, miR-138-5p, miR-206, miR-23c and miR-223, had potential binding sites with KLF3-AS1. Compared to those in cells transfected with control vector, miR-185-5p and miR-206 were declined after overexpression of KLF3-AS1, especially, miR-206 was the most notably decreased one, which was chosen for the following experiments (Fig. 5A). With the transfection of miR-206 mimic, the luciferase activity in wt-KLF3-AS1 and wt-USP22 groups was both repressed, in comparison with that in mut-KLF3-AS1 and mut-USP22 groups, respectively (Fig. 5B, C). To further assess the interaction among these three molecules, Ago2-RIP assays were carried out. Endogenous KLF3-AS1, miR-206 and USP22 pulled-down by Ago2 antibody were enriched, compared to that of anti-IgG group (Fig. 5D). Moreover, the enrichment of KLF3-AS1 and USP22 pulled-down by Ago2 antibody were both upregulated in KLF3-AS1 transfected cells (Fig. 5E). Overexpression of KLF3-AS1 upregulated KLF3-AS1 and USP22 mRNA levels, but downregulated miR-206 expression (Fig. 5F). miR-206 mimics could counteract the upregulated USP22 mRNA and protein expressions induced by overexpression of KLF3-AS1 (Fig. 5G, H). Taken together, the discoveries indicated that KLF3-AS1 acted as a ceRNA and sponged miR-206 to upregulate USP22.

### KLF3-AS1/miR-206 axis regulated cerebral I/R injury through Sirt1

**Fig. 6:**
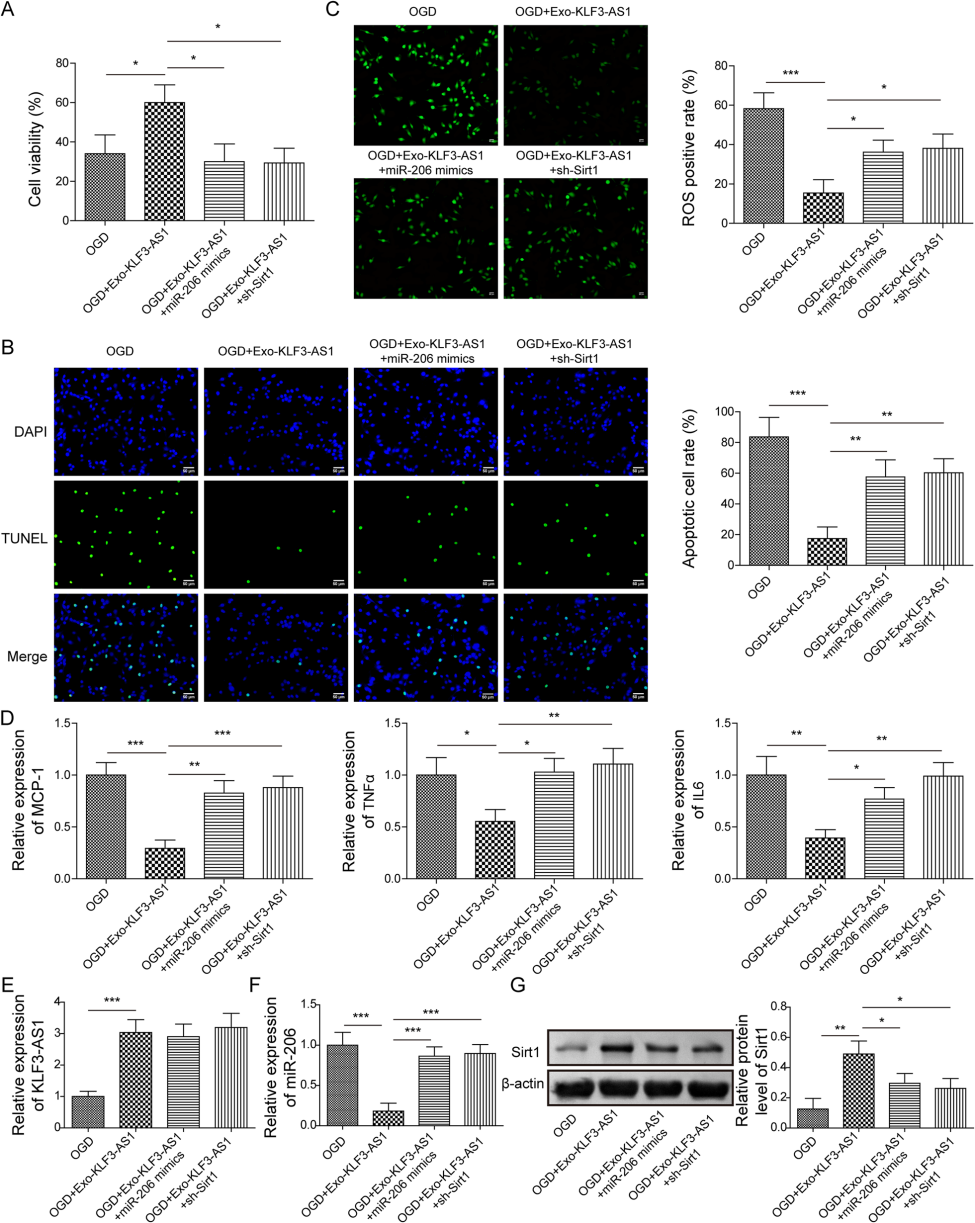
The effect of Exos-KLF3-AS1 on cerebral I/R injury could be overturned by miR-206 overexpression or Sirt1 knockdown. Cells transfected with miR-206 mimic or sh-Sirt1 were subjected to OGD, prior to supplemented with Exos-KLF3-AS1. **A** Cell viability was assessed. **B** TUNEL-positive cells. **C** The production of ROS. **D** The mRNA levels of MCP-1, TNFα and IL-6. **E**, **F** qRT-PCR detection of KLF3-AS1 (**E**) and miR-206 (**F**). **G** Sirt1 protein was determined. Values were expressed as mean ± SD of three separate determinations in the in vitro assays. *P < 0.05, **P < 0.01, ***P < 0.001

**Fig. 7:**
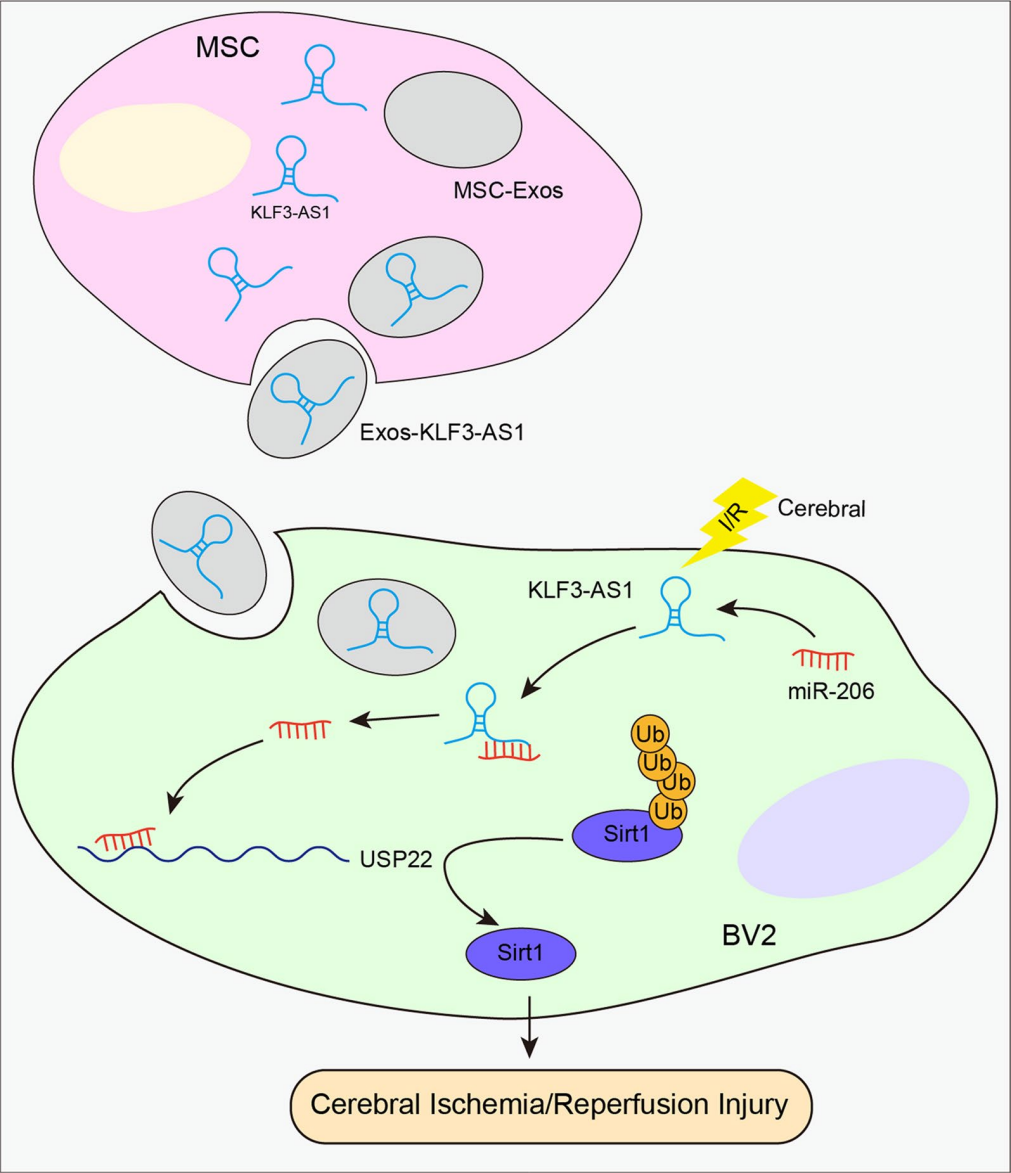
A schematic graph for drawing the role of KLF3-AS1 in cerebral I/R injury

We further explored whether exosomal KLF3-AS1 affected cerebral I/R damage through modulating Sirt1 via targeting miR-206. Exos-KLF3-AS1 alleviated OGD-induced cell injury, overexpression of miR-206 or silencing of Sirt1 reversed the effect (Fig. 6A). Exos-KLF3-AS1 inhibited cell apoptosis, ROS production and inflammation during OGD exposure, whereas overexpression of miR-206 or silencing of Sirt1 removed the benefit roles of Exos-KLF3-AS1 (Fig. 6B-D). Exos-KLF3-AS1 treatment increased KLF3-AS1 expression, but overexpression of miR-206 or silencing of Sirt1 did not affect its expression (Fig. 6E). Exos-KLF3-AS1 decreased miR-206 expression, which could be reversed by overexpression of miR-206 but not affected after silencing of Sirt1 (Fig. 6F). Exos-KLF3-AS1 increased Sirt1 protein level, whereas overexpression of miR-206 or silencing of Sirt1 could upregulated its expression (Fig. 6G). Taken together, Exos-KLF3-AS1 from BMSC upregulated Sirt1 to inhibit cerebral I/R injury via targeting miR-206 (Fig. 7).

## Discussion

Recently, BMSC has presented augmented protection for treating cerebral I/R injury (Zeng et al. 2020). MSC-derived exosomes can mediate the function of MSC by delivering many kinds of mediators, such as lncRNAs and microRNAs (Moghadasi et al. 2021). In addition, lncRNAs have been proven to participate in the pathogenesis of cerebral I/R injury (Vasudeva et al. 2021). Here, we investigated the role of BMSC-Exos KLF3-AS1 in apoptosis and inflammatory injury in cerebral I/R. Collectively, our data revealed BMSC-Exos KLF3-AS1 indeed alleviated cerebral I/R damage through promoting the stability of Sirt1 via KLF3-AS1/miR-206/USP22 network.

KLF3-AS1 delivered by BMSC-extracellular vesicles attenuates neurological deficits after subarachnoid hemorrhage (Cheng et al. 2022). KLF3-AS1 upregulation in BMSC-Exos results in the attenuated myocardial infarction and the repressed cell apoptosis (Mao et al. 2019). Moreover, BMSC-Exos KLF3-AS1 suppresses apoptosis and inflammation in endothelial cells challenged by high glucose (Han et al. 2022). Here, we found that BMSC-Exos KLF3-AS1 reduced cerebral infarction and improved neurological function in MCAO mice. In OGD-treated BV-2 cells, BMSC-Exos KLF3-AS1 promoted cell viability, repressed apoptosis and inflammatory injury. The results provide the first evidence that BMSC-Exos KLF3-AS1 ameliorates cerebral I/R injury in vivo and in vitro, to our knowledge.

KLF3-AS1 in exosomes secreted from MSC can regulate Sirt1 so as to attenuate myocardial infarction (Mao et al. 2019). Sirt1 has been verified to play an essential role in cerebral I/R injury (Xian et al. 2019; Zhou et al. 2021). Sirt1 expression is downregulated in OGD-treated SH-SY5Y cells and cerebral I/R rats, and cerebral I/R injury is enhanced by miR-7-5p via degrading Sirt1 mRNA (Zhao and Wang 2020). We discovered that BMSC-Exos KLF3-AS1 elevated the downregulated Sirt1 induced by cerebral I/R damage in vitro and in vivo. KLF3-AS1 overexpression upregulated Sirt1 protein; furthermore, KLF3-AS1 overexpression prolonged the half-life of Sirt1 protein under cycloheximide treatment, as well as protected Sirt1 protein from proteasome degradation. As far as we know, the research is the first to identify that KLF3-AS1 facilitates Sirt1 protein expression via inhibiting Sirt1 degradation.

The decreased Sirt1 expression induced by the promoted Sirt1 ubiquitination can facilitate neuronal apoptosis in cerebral I/R (Xue et al. 2022). We validated that overexpression of KLF3-AS1 promoted the deubiquitination of Sirt1 protein. USP22 can modulate the process of deubiquitination to stabilize Sirt1 (Lin et al. 2012). Furthermore, USP22 stabilizes Sirt1 expression via regulating deubiquitination to protect against myocardial I/R damage (Ma et al. 2020). Here, USP22 was upregulated in BV-2 cells with overexpression of KLF3-AS1. Moreover, silencing of USP22 reversed the promoted deubiquitinating of Sirt1 protein caused by overexpression of KLF3-AS1. Besides, the direct interaction of USP22 and Sirt1 was validated by co-immunoprecipitation, and the result was consistent with the precious study (Ma et al. 2020). Importantly, we are the first to validate that the deubiquitination of Sirt1 protein can be promoted by KLF3-AS1 via inducing USP22.

LncRNA-microRNA-mRNA has been validated to form a complex regulatory network which plays a critical role in I/R damage (Gong et al. 2020). For example, lncRNA CEBPA-AS1 sponges miR-340-5p to alleviate cerebral I/R damage through modulating downstream pathway (Tu et al. 2022). Bioinformatics prediction presents potential binding sites between KLF3-AS1 and miR-206. In subarachnoid hemorrhage induced cerebral injury, miR-206-knockdown exosomes derived from MSC exerts a neuroprotective function (Zhao et al. 2019). In osteoarthritis, MSC-derived exosomes inhibit chondrocytes apoptosis via KLF3-AS1/miR-206 axis (Liu et al. 2018). Here, we validated that KLF3-AS1 sponged miR-206 to downregulate its level, thereby upregulating USP22. Specifically, overexpression of miR-206 or silencing of Sirt1 exhibited a reversed effect to that induced by BMSC-Exos KLF3-AS1, suggesting that the effect of BMSC-Exos KLF3-AS1 might converge on miR-206/Sirt1 axis. Our study demonstrated that KLF3-AS1 was a modulator of BMSC exosomes and acted as a ceRNA of miR-206 to upregulate Sirt1. In the present study, BV2 cells were selected for the in vitro experiments (Zhou et al. 2022a, b). Further in vitro studies in a neuronal cell line or primary neuronal cultures are warranted to validate the effect of BMSC-Exos KLF3-AS1 on cerebral I/R injury. In addition, young adult male mice were employed for in vivo experiments, further in vivo researches should be carried out in mice with different gender and age.

## Conclusion

These findings provided evidence that BMSC-Exos KLF3-AS1 improved inflammatory injury and hindered apoptosis caused by cerebral I/R through promoting Sirt1 stability via KLF3-AS1/miR-206/USP22 network. Thus, the present results showed that BMSC-Exos KLF3-AS1 might present a prospective therapeutic target for treating cerebral I/R.

## Supplementary Information

**Additional file 1: Fig. S1.** Identification of BMSC and BMSC-Exos. (A) BMSC markers (HLA-DR, CD105, CD90, CD45 and CD34) tested using flow cytometry. (B) Representative BMSC-Exos morphology obtained by TEM. (C) NTA was employed for detecting the diameter distribution of BMSC-Exos. (D) The detection of BMSC-Exos biomarkers using western blot. (E) The expression of KLF3-AS1. (F and G) Exosomes were isolated from BMSC that transfected with KLF3-AS1 overexpression vector or its corresponding empty vector, and the expression of KLF3-AS1 in BMSC and exosomes was measured by qRT-PCR. Values were expressed as mean ± SD of three separate determinations in the in vitro assays. *P < 0.05, **P < 0.01, ***P < 0.001.

## Data Availability

All data generated or analyzed during this study are included in this published article.

## Abbreviations

BMSC: Bone marrow mesenchymal stem cell
MSC: Mesenchymal stem cells
Exos: Exosomes
lncRNAs: Long non-coding RNAs
miR: MicroRNA
I/R: Ischemia/reperfusion
USP22: Ubiquitin specific peptidase 22
Sirt1: Silent mating type information regulation 2 homolog 1
NAD: Nicotine adenine dinucleotide
ceRNA: Competing endogenous RNA
MCAO: Middle cerebral artery occlusion
OGD: Oxygen–glucose deprivation
mNSS: Modified neurological severity score
ROS: Reactive oxygen species
TNFα: Tumor necrosis factor alpha
IL-6: Interleukin 6
MCP-1: Monocyte chemotactic protein-1
