# Supplementary figures and images for: Bone marrow mesenchymal stem cell-derived exosomal lncRNA KLF3-AS1 stabilizes Sirt1 protein to improve cerebral ischemia/reperfusion injury via miR-206/USP22 axis
Source: Mol Med. 2023 Jan 10;29:3. doi: 10.1186/s10020-022-00595-1 (PMC9830826; doi:10.1186/s10020-022-00595-1)

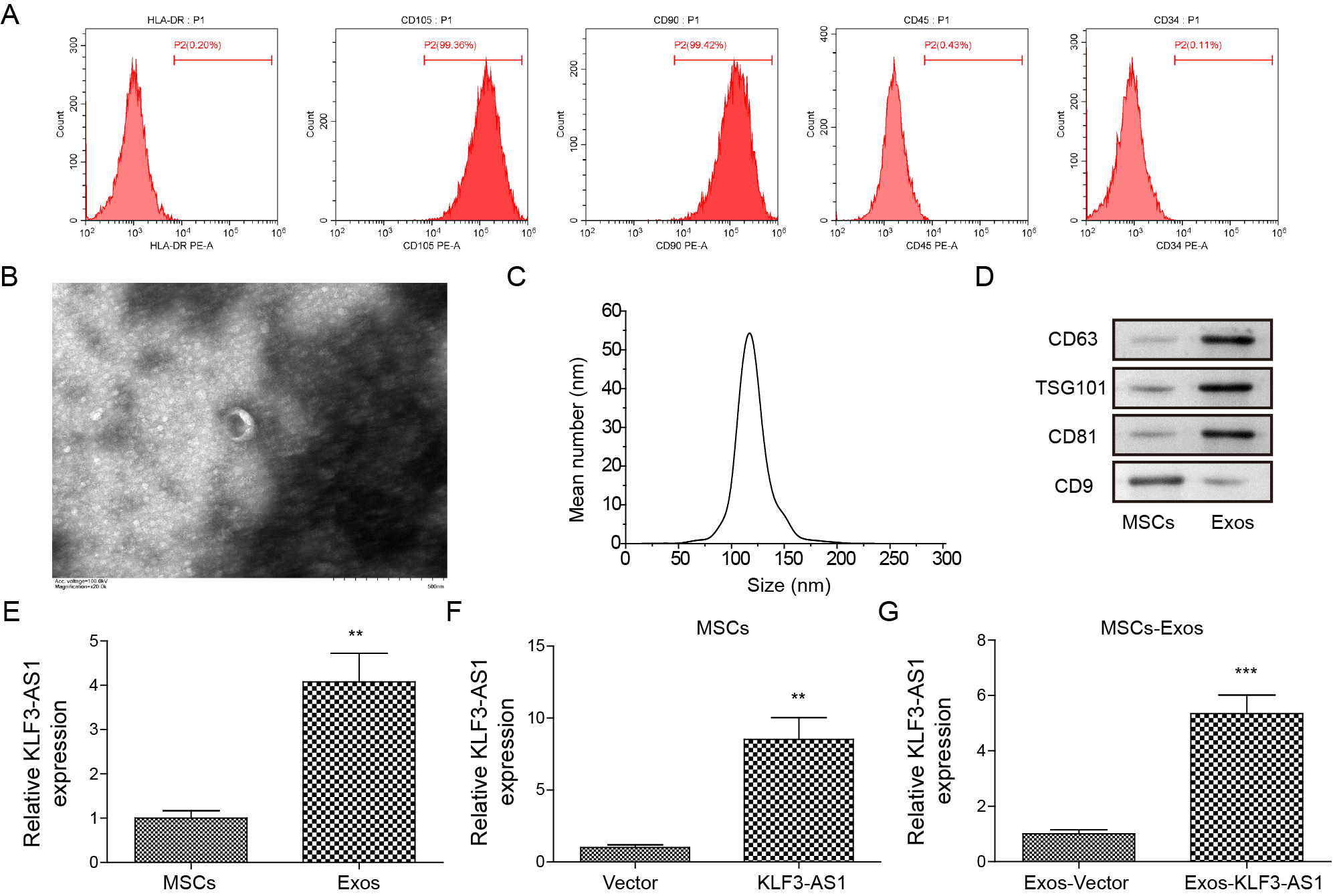

Supplement: Supplementary file 1 — Additional file 1: Fig. S1. Identification of BMSC and BMSC-Exos. (A) BMSC markers (HLA-DR, CD105, CD90, CD45 and CD34) tested using flow cytometry. (B) Representative BMSC-Exos morphology obtained by TEM. (C) NTA was employed for detecting the diameter distribution of BMSC-Exos. (D) The detection of BMSC-Exos biomarkers using western blot. (E) The expression of KLF3-AS1. (F and G) Exosomes were isolated from BMSC that transfected with KLF3-AS1 overexpression vector or its corresponding empty vector, and the expression of KLF3-AS1 in BMSC and exosomes was measured by qRT-PCR. Values were expressed as mean ± SD of three separate determinations in the in vitro assays. *P < 0.05, **P < 0.01, ***P < 0.001. [file 10020_2022_595_MOESM1_ESM.tif]
